# The composite risk index based on frailty predicts postoperative complications in older patients recovering from elective digestive tract surgery: a retrospective cohort study

**DOI:** 10.1186/s12871-021-01549-6

**Published:** 2022-01-03

**Authors:** Chun-Qing Li, Chen Zhang, Fan Yu, Xue-Ying Li, Dong-Xin Wang

**Affiliations:** 1 Department of Anesthesiology and Critical Care Medicine, Peking University First Hospital, No. 8, Xishiku Street, Beijing, 100034 China; 2 Department of Biostatistics, Peking University First Hospital, Beijing, China; 3 Outcomes Research Consortium, Cleveland, OH USA

**Keywords:** Older patient, Frailty, Malnutrition, Digestive system surgical procedures, Postoperative complications

## Abstract

**Background:**

Limitations exist in available studies investigating effect of preoperative frailty on postoperative outcomes. This study was designed to analyze the association between composite risk index, an accumulation of preoperative frailty deficits, and the risk of postoperative complications in older patients recovering from elective digestive tract surgery.

**Methods:**

This was a retrospective cohort study. Baseline and perioperative data of older patients (age ≥ 65 years) who underwent elective digestive tract surgery from January 1, 2017 to December 31, 2018 were collected. The severity of frailty was assessed with the composite risk index, a composite of frailty deficits including modified frailty index. The primary endpoint was the occurrence of postoperative complications during hospital stay. The association between the composite risk index and the risk of postoperative complications was assessed with a multivariable logistic regression model.

**Results:**

A total of 923 patients were included. Of these, 27.8% (257) developed postoperative complications. Four frailty deficits, i.e., modified frailty index ≥0.27, malnutrition, hemoglobin < 90 g/L, and albumin ≤30 g/L, were combined to generate a composite risk index. Multivariable analysis showed that, when compared with patients with composite risk index of 0, the odds ratios (95% confidence intervals) were 2.408 (1.714–3.383, *P* <  0.001) for those with a composite risk index of 1, 3.235 (1.985–5.272, *P* <  0.001) for those with a composite risk index of 2, and 9.227 (3.568–23.86, *P* <  0.001) for those with composite risk index of 3 or above. The area under receiver-operator characteristic curve to predict postoperative complications was 0.653 (95% confidence interval 0.613–0.694, *P* <  0.001) for composite risk index compared with 0.622 (0.581–0.663, *P* <  0.001) for modified frailty index.

**Conclusion:**

For older patients following elective digestive tract surgery, high preoperative composite risk index, a combination of frailty deficits, was independently associated with an increased risk of postoperative complications.

**Supplementary Information:**

The online version contains supplementary material available at 10.1186/s12871-021-01549-6.

## Background

Frailty is a geriatric syndrome characterized by declined physiologic reserve and impaired capacity to maintain homeostasis [1, 2]. The etiology of frailty is multifactorial but may include the accumulation of degenerative changes and disease-associated deficits across multiple systems, involving functional, medical, nutritional, psychosocial, and cognitive domains, all of which increase vulnerability to stress. In particular, the progressive nutritional and medical deteriorations caused by new-onset diseases such as cancer contribute to the development or aggravation of frailty. With the accelerated aging process, the proportion of older people (aged ≥65 years) in the Chinese population is rapidly increasing [3]. It is estimated that more than 50% of older people will receive at least one surgery during their remaining lifespan [4]. However, frailty among older patients not only decreases their resilience to surgical trauma, but also delays their postoperative recovery. This brings a great challenge to the perioperative care providers.

It is recommended that frailty assessment should be routinely performed for older patients before surgery [5, 6]. Numerous instruments, including the modified frailty index (mFI), have been developed to assess frailty [7–15]. Current evidence indicates that the presence of frailty is associated with increased perioperative morbidity and mortality [7, 10, 14, 16–24]. However, limitations exist in the available results. For example, as one of the most frequently used preoperative frailty scales, the mFI does not include recent changes induced by surgical diseases for which surgeries are planning to be performed [7, 16–24]. These changes, such as loss of body weight, low albumin, and anemia, may also aggravate frailty and be associated with worse outcomes [25–28]. Additionally, there are also studies reporting “negative” results [29–31]. Therefore, further studies are required to improve the method for frailty evaluation and to clarify the correlation between preoperative frailty and postoperative outcomes.

We hypothesized that a higher preoperative composite risk index, accumulation of frailty deficits including the mFI, was associated with an increased risk of adverse postoperative outcomes in older patients. The primary purpose of this study was to analyze the relationship between the composite risk index and the occurrence of postoperative complications (POCs) in older patients recovering from elective digestive tract surgery.

## Methods

### Study design

This retrospective cohort study was performed in Peking University First Hospital, a tertiary general hospital in Beijing, China. The study protocol was approved by the Biomedical Research Ethics Committee of Peking University First Hospital (2019[296], Beijing, China). As the study was purely observational and no patient follow-up was performed, the Ethics Committee agreed to waive the written informed consent from patients. All personal data were kept strictly confidential.

### Patient selection

Older patients (age ≥ 65 years) who underwent elective digestive system surgery from January 1, 2017 to December 30, 2018 in Beijing University First Hospital were screened utilizing the medical records system. Patients who met the following criteria were excluded: (1) underwent combined surgery; (2) incomplete or missing perioperative data.

### Data collection

All data were extracted from the electronic medical records system of Peking University First Hospital. To eliminate the risk of diagnostic bias, covariates and outcomes were separately collected by different investigators (CZ and FY) who were strictly trained and blinded to the purpose of the study.

Baseline data were collected and included demographic characteristics (age, sex, and body mass index), surgical diagnosis, comorbidity, body weight change in the last 3–6 months, history of smoking and drinking [32], and main laboratory test results. Physical status was classified according to the American Society of Anesthesiologists (ASA) Classification. The 11 components of the mFI were collected according to the National Surgical Quality Improvement Program definitions (Supplementary Table 1); each item was assigned the same weight of 1 point. The mFI score was calculated by summarizing the total points and then dividing them by 11. The resulting index ranges from 0 to 1.0, with a higher score indicating more severe frailty [7]. Nutritional status was assessed according to the National Institute for Health and Clinical Excellence (NICE) guidance for “Nutrition support for adults: oral nutrition support, enteral tube feeding and parenteral nutrition (2006)”, which defines malnutrition as meeting any of the following: (1) a body mass index of less than 18.5 kg/m^2^; (2) unintentional weight loss of greater than 10% within the last 3–6 months; or (3) a body mass index of less than 20 kg/m^2^ and unintentional weight loss of greater than 5% within the last 3–6 months [33].

Intraoperative data were also collected and included type and duration of surgery, type of anesthesia, the seniority of anesthesiologists, estimated blood loss, and intraoperative blood transfusion. The type of surgery was stratified into five categories according to the Operative Stress Score, i.e., very low stress, low stress, moderate stress, high stress, and very high stress (Supplementary Table 2) [34]. If more than one surgical procedure (such as unplanned reoperation for bleeding or other complications) was performed during hospitalization, only the first procedure was taken into analysis.

The primary outcome was the development of POCs during hospital stay. POCs were defined as any deviation from a normal postoperative course that was harmful to patients’ recovery and required different levels of therapeutic intervention, i.e., graded II or higher according to the Clavien**-**Dindo classification (Supplementary Table 3) [35]. If multiple complications occurred in a patient, only the most severe one was analyzed. Secondary outcomes included the intensive care unit (ICU) admission after surgery, length of ICU stay, unplanned reintubation/reoperation, total length of hospital stay and length of hospital stay after surgery, and adverse discharge destination.

### Statistical analysis

The baseline and perioperative data were compared between patients with POCs and those without. Continuous variables were analyzed with independent samples *t* tests or Mann-Whitney *U* tests. Categorical variables were analyzed using chi-square tests, continuity-corrected chi-square tests, or Fisher’s exact tests. Time-to-event variables were analyzed with Kaplan-Meier survival analyses, with the differences between groups tested with Log-Rank tests. Univariable logistic regression analyses were used to screen potential risk factors of POCs. Independent variables with *P* values < 0.20 in univariate analysis and those that were considered clinically important were included in a multivariable logistic regression model to identify independent predictors of POCs with the Wald (backward) method.

According to the results of primary multivariable analysis, we divided the mFI dichotomously and selected other independent predictors of POCs that reflected the frailty features of the study cohort. We combined these parameters to generate a composite risk index. We then performed another multivariable logistic regression analysis to evaluate the effects of the composite risk index in predicting POCs after adjustment for confounding factors.

We also compared postoperative outcomes among patients with different mFI or composite risk index scores. Outcomes of two groups were compared as above. For outcomes of three or more groups, categorical variables were compared with the Chi-squared tests or Fisher’s exact tests and post hoc Chi-squared tests or Fisher’s exact tests. Time-to-event variables were analyzed with Kaplan-Meier survival analyses and Log-Rank tests. The predictive performances of the mFI and composite risk index in predicting POCs were assessed using the receiver-operating characteristic (ROC) curve analysis. The area under the curve and 95% confidence interval (CI) were provided to describe their discriminative power.

Two-tailed *P* values of < 0.05 were considered statistically significant. Bonferroni correction was performed for multiple comparisons. Statistical analysis was performed with the SPSS version 25.0 (IBM SPSS, Inc., Chicago, IL).

According to the “ten events per variable” rule and the number of independent variables (15 or 12) included in the multivariable logistic regression models, the number of patients with primary outcome (257) was sufficient [36], although estimation of sample size was not performed in advance. Therefore, the sample size of participants (923) included in our study was adequate and could guarantee the stability of the regression estimates.

## Results

### Patients

**Fig. 1:**
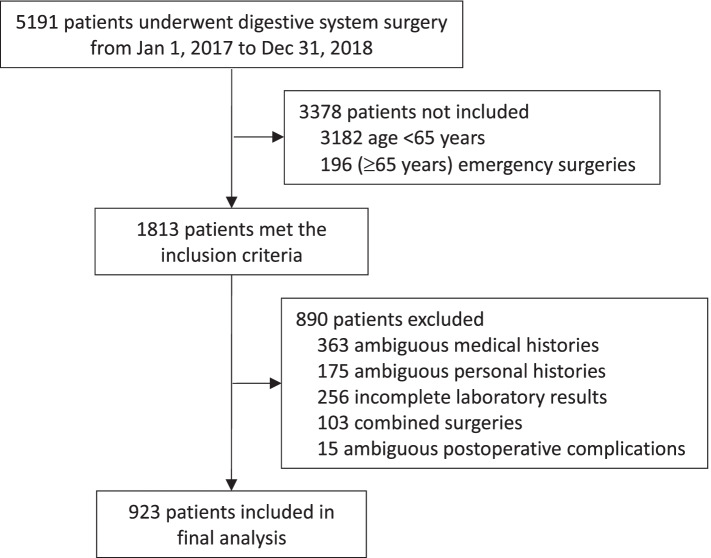
Flowchart of the study

From January 1, 2017 to December 31, 2018, a total of 5191 patients underwent digestive tract surgery. Of these, 3378 patients were excluded because they did not meet the inclusion criteria (age < 65 years or emergency surgeries); 890 patients were excluded because they met the criteria of exclusion (ambiguous medical or personal histories, incomplete preoperative laboratory test results, combined surgeries, or missing data of postoperative complications). At last, 923 patients were included in the final analysis (Fig. 1).

The study population had a mean age of 73.5 years; 37.6% (347/923) were female. Of the included patients, 23.8% (220) had a mFI of 0.00, 30.8% (284) a mFI of 0.09, 21.0% (194) a mFI of 0.18, 12.7% (117) a mFI of 0.27, 8.1% (75) a mFI of 0.36, and 3.6% (33) a mFI of 0.45 or above, and 23.0% (212) met the criteria of malnutrition. During surgery, 6.1% (56) underwent low-stress procedures, 28.8% (266) moderate-stress procedures, 57.0% (526) high-stress procedures, and 8.1% (75) very high-stress procedures. After surgery, 27.8% (257) developed complications, 25.6% (236) were admitted to the ICU; the median length of hospital was 16.0 days (Table 1; Supplementary Tables 1, 2, 3 and 4). Baseline and intraoperative data according to modified frailty index and composite risk index are listed in Supplementary Table 5.

**Table 1:** Baseline and perioperative data

	All patients	Without postoperative complications	With postoperative complications	*P* value
	(*n* = 923)	(*n* = 666)	(*n* = 257)	
**Demographic data**				
Age, year	73.5 ± 6.2	73.3 ± 6.1	74.1 ± 6.3	0.092
Female gender	347 (37.6%)	243 (36.5%)	104 (40.5%)	0.263
Body mass index				**0.049**
< 18.5 kg/m^2^	58 (6.3%)	33 (5.0%)	25 (9.7%)	
18.5–23.9 kg/m^2^	467 (50.6%)	347 (52.1%)	120 (46.7%)	
24–27.9 kg/m^2^	315 (34.1%)	227 (34.1%)	88 (34.2%)	
≥ 28 kg/m^2^	83 (9.0%)	59 (8.9%)	24 (9.3%)	
**General status**				
ASA class				**< 0.001**
I	7 (0.8%)	5 (0.8%)	2 (0.8%)	
II	537 (58.2%)	430 (64.6%)	107 (41.6%)	
III	361 (39.1%)	225 (33.8%)	136 (52.9%)	
IV	18 (2.0%)	6 (0.9%)	12 (4.7%)	
Modified frailty index				**< 0.001**
0.00	220 (23.8%)	177 (26.6%)	43 (16.7%)	
0.09	284 (30.8%)	221 (33.2%)	63 (24.5%)	
0.18	194 (21.0%)	139 (20.9%)	55 (21.4%)	
0.27	117 (12.7%)	75 (11.3%)	42 (16.3%)	
0.36	75 (8.1%)	41 (6.2%)	34 (13.2%)	
0.45	23 (2.5%)	9 (1.4%)	14 (5.4%)	
0.55	7 (0.8%)	3 (0.5%)	4 (1.6%)	
0.64	3 (0.3%)	1 (0.2%)	2 (0.8%)	
Malnutrition^a^	212 (23.0%)	128 (19.2%)	84 (32.7%)	**< 0.001**
**Comorbidities and history** ^b^				
Asthma	19 (2.1%)	13 (2.0%)	6 (2.3%)	0.714
Obstructive sleep apnea ^c^	42 (4.6%)	19 (2.9%)	23 (8.9%)	**< 0.001**
Severe arrhythmia ^d^	77 (8.3%)	48 (7.2%)	29 (11.3%)	**0.045**
Other cardiac diseases ^e^	24 (2.6%)	15 (2.3%)	9 (3.5%)	0.285
Mental disorders ^f^	21 (2.3%)	14 (2.1%)	7 (2.7%)	0.570
Major neurodegenerative diseases ^g^	16 (1.7%)	7 (1.1%)	9 (3.5%)	**0.023**
Visual/hearing impairment	31 (3.4%)	18 (2.7%)	13 (5.1%)	0.075
Chronic renal insufficiency ^h^	31 (3.4%)	17 (2.6%)	14 (5.4%)	**0.029**
Chronic hepatic dysfunction ^i^	49 (5.2%)	27 (4.1%)	22 (8.2%)	**0.006**
Hyper−/hypothyroidism	19 (2.1%)	14 (2.1%)	5 (1.9%)	0.881
Chronic corticosteroid therapy ^j^	26 (2.8%)	16 (2.4%)	10 (3.9%)	0.220
Malignant tumor	741 (80.3%)	514 (77.2%)	227 (88.3%)	**< 0.001**
Current smoker/quit ≤4 weeks ^k^	134 (14.5%)	93 (14.0%)	41 (16.0%)	0.442
Current alcoholism/quit ≤4 weeks ^l^	42 (4.6%)	28 (4.2%)	14 (5.4%)	0.417
**Laboratory tests**				
Hemoglobin < 90 g/L	82 (8.9%)	41 (6.2%)	41 (16.0%)	**< 0.001**
Albumin ≤30 g/L	44 (4.8%)	20 (3.0%)	24 (9.3%)	**< 0.001**
Na^+^ < 135.0 mmol/L	116 (12.6%)	81 (12.2%)	35 (13.6%)	0.550
Ca^++^ < 2.1 mmol/L	32 (3.5%)	21 (3.2%)	11 (4.3%)	0.402
K^+^ < 3.5 or > 5.5 mmol/L	99 (10.7%)	70 (10.5%)	29 (11.3%)	0.734
**Composite risk index** ^m^				**< 0.001**
0	502 (54.4%)	413 (62.0%)	89 (34.6%)	
1	306 (33.2%)	196 (29.4%)	110 (42.8%)	
2	92 (10.0%)	50 (7.5%)	42 (16.3%)	
≥ 3	23 (2.5%)	7 (1.1%)	16 (6.2%)	
**Intraoperative data**				
Type of surgery				**< 0.001**
Simple general surgeries ^n^	80 (8.7%)	77 (11.6%)	3 (1.2%)	
Gastric	154 (16.7%)	100 (15.0%)	54 (21.0%)	
Intestinal	556 (60.2%)	414 (62.2%)	142 (55.3%)	
Hepatopancreatobiliary	133 (14.4%)	75 (11.3%)	58 (22.6%)	
Surgery by Operative Stress Score ^o^				**< 0.001**
Low stress	56 (6.1%)	55 (8.3%)	1 (0.4%)	
Moderate stress	266 (28.8%)	215 (32.3%)	51 (19.8%)	
High stress	526 (57.0%)	357 (53.6%)	169 (65.8%)	
Very high stress	75 (8.1%)	39 (5.9%)	36 (14.0%)	
Duration of surgery, hour	3.2 (2.4, 4.3)	3.0 (2.3, 4.0)	3.6 (2.6, 5.0)	**< 0.001**
Type of anesthesia				0.346
General	448 (48.5%)	321 (48.2%)	127 (49.4%)	
Combined PNB-general	438 (47.5%)	322 (48.3%)	116 (45.1%)	
Combined epidural-general	32 (3.5%)	19 (2.9%)	13 (5.1%)	
Neuraxial	5 (0.5%)	4 (0.6%)	1 (0.4%)	
Seniority of anesthesiologists				0.490
< 5 years	259 (28.0%)	194 (29.1%)	65 (25.3%)	
5 to 10 years	175 (19.0%)	123 (18.5%)	52 (20.2%)	
> 10 years	489 (53.0%)	349 (52.4%)	140 (54.5%)	
Blood transfusion	64 (6.9%)	33 (5.0%)	31 (12.1%)	**< 0.001**
Estimated blood loss, ml	100 (50, 200)	50 (50, 150)	100 (50, 200)	**< 0.001**
**Postoperative data**				
Postoperative complications ^p^	257 (27.8%)	–	257 (100.0%)	–
Clavien-Dindo classification ^q^				–
Grade II	160 (17.3%)	–	160 (62.3%)	
Grade III	33 (3.6%)	–	33 (12.8%)	
Grade IV	57 (6.2%)	–	57 (22.2%)	
Grade V	7 (0.8%)	–	7 (2.7%)	
ICU admission	236 (25.6%)	106 (15.9%)	130 (50.6%)	**< 0.001**
LOS in ICU, hour	24.0 (18.0, 48.0)	20.0 (16.0, 24.0)	41.0 (20.0, 91.0)	**< 0.001**
Unplanned reintubation	12 (1.3%)	0 (0%)	12 (4.7%)	**< 0.001**
Unplanned reoperation	28 (3.0%)	0 (0%)	28 (10.9%)	**< 0.001**
Hospital LOS, day	16.0 (14.0, 21.0)	15.0 (13.0, 19.0)	21.0 (16.0, 29.0)	**< 0.001**
Hospital LOS after surgery, day	10.0 (8.0, 12.0)	9.0 (7.0, 10.0)	13.0 (11.0, 20.0)	**< 0.001**
Adverse discharge destination ^r^	15 (1.6%)	0 (0%)	15 (5.8%)	**< 0.001**

Data are n (%), mean ± SD, or median (interquartile range). *P* values in bold indicate < 0.05

*ASA* American Society of Anesthesiologists, *PNB* peripheral nerve block, *ICU* intensive care unit, *LOS* length of stay

^a^ Defined by any of the following: (1) a body mass index of less than 18.5 kg/m^2^; (2) unintentional weight loss of greater than 10% within the last 3–6 months; or (3) a body mass index of less than 20 kg/m^2^ and unintentional weight loss of greater than 5% within the last 3–6 months [33]

^b^ Data on 11 items of the modified frailty index are presented in Supplementary Table 1

^c^ Diagnosed by previous polysomnography, or history inquiry and physical examination, and/or STOP-Bang/Berlin questionnaire

^d^ Include atrial fibrillation, frequent (> 6 beats/min) or multifocal ventricular premature beat, paroxysmal supraventricular tachycardia, second/third-degree atrioventricular block, and sick sinus syndrome

^e^ Include congenital heart disease, cardiomyopathy, and valvular heart disease

^f^ Include diagnosed depression, anxiety, schizophrenia, phobia, and hallucination

^g^ Include Alzheimer’s disease, Parkinson’s disease, and dementia

^h^ Refers to estimated glomerular filtration rate < 45 ml/min/1.73 m^2^ or on dialysis [37]. The CKD-EPI equation was adopted to calculate the estimated glomerular filtration rate [38]

^i^ Defined as Child-Pugh class B and C

^j^ With a duration of > 1 month

^k^ Smoking refers to daily smoking of cigarettes up to half a pack for at least two years

^l^ Alcoholism refers to ethanol consumption ≥40 g/d for men and ≥ 20 g/d for women, lasting for more than 5 years. Ethanol (g) = alcohol consumption (ml) × ethanol content (%) × 0.8 [32]

^m^ A composite of four items, i.e., modified frailty index ≥0.27, malnutrition [33], moderate or severe anemia (hemoglobin < 90 g/L), and severe hypoalbuminemia (albumin ≤30 g/L). Each item was assigned the same weight of 1 point

^n^ Refers to low-risk and 23-h-stay operations including hernia repair, laparoscopic cholecystectomy, appendectomy, and hepatic cyst fenestration

^o^ Stratified into five categories of physiologic stress, i.e., very low stress, low stress, moderate stress, high stress, and very high stress [34]. Also see Supplementary Table 2

^p^ Indicate those of Clavien-Dindo grade II or higher. Also see Supplementary Table 4

^q^ Clavien-Dindo classification of postoperative complications [35]

^r^ Defined as discharge to destinations other than home (e.g., a long- or short-term care facility)

### Association between mFI and POCs

**Fig. 2:**
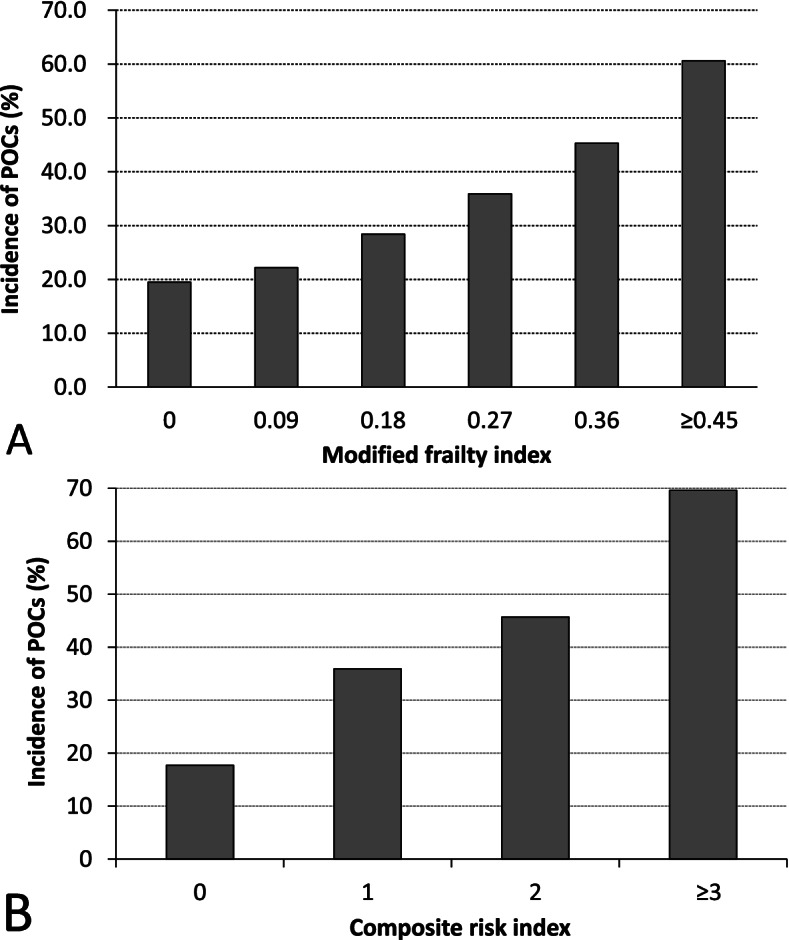
Incidence of postoperative complications in subgroups according to modified frailty index (**A**) and composite risk index (**B**). When compared with patients with mFI = 0, mFI = 0.09: *P* = 0.471, mFI = 0.18: *P* = 0.035, mFI = 0.27: *P* = 0.001, mFI = 0.36: *P* <  0.001, and mFI ≥ 0.45: *P* <  0.001 (chi-square tests; *P* <  0.003 was considered statistically significant after Bonferroni correction). When compared with patients with composite risk index of 0, composite risk index of 1: *P* <  0.001, composite risk index of 2: *P* < 0.001, composite risk index of 3 or above: *P* < 0.001 (chi-square tests; *P* < 0.008 was considered statistically significant after Bonferroni correction). Abbreviations: *POCs* Postoperative complications

As the mFI score increased from 0 to 0.45 or above, the incidence of POCs in the six mFI subgroups increased accordingly (Fig. 2A). Univariable analyses identified 17 factors (excluding composite risk index) with *P* <  0.20. Among these, high mFI was associated with an increased risk of POCs (Supplementary Table 6).

Fifteen factors were included in the multivariable logistic model. After correction for confounding factors, high mFI remained to be significantly associated with an increased risk of POCs; when compared with patients with mFI of 0, the odd ratios (ORs) were 1.113 (95% confidential interval [CI] 0.703–1.764, *P* = 0.648) for those with mFI of 0.09, 1.519 (95% CI 0.931–2.476, *P* = 0.094) for those with mFI of 0.018, 2.250 (95% CI 1.316–3.848, *P* = 0.003) for those with mFI of 0.27, 3.663 (95% CI 1.996–6.721, *P* <  0.001) for those with mFI of 0.36, and 5.495 (95% CI 2.396–12.60, *P* <  0.001) for those with mFI of 0.45 or above (Table 2). Among other independent factors, malnutrition (OR 1.522, 95% CI 1.068–2.170, *P* = 0.020), hemoglobin < 90 g/L (OR 1.794, 95% CI 1.072–3.001, *P* = 0.026), albumin ≤30 g/L (OR 2.051, 95% CI 1.032–4.078, *P* = 0.040), obstructive sleep apnea (OR 2.776, 95% CI 1.379–5.586, *P* = 0.004), surgery with moderate or higher stress (compared with low-stress procedures, moderate-stress procedures: OR 10.34, 95% CI 1.371–78.04, *P* = 0.023; high-stress procedures: OR 15.86, 95% CI 2.106–119.4, *P* = 0.007; very high-stress procedures: OR 22.40, 95% CI 2.755–182.2, *P* = 0.004), and long-duration surgery (per hour: OR 1.127, 95% CI 1.003–1.266, *P* = 0.045) were also associated with increased risk of POCs (Table 2).

**Table 2:** Predictors of postoperative complications

Variables	Univariable analyses		Multivariable analysis ^a^	
	Odds ratio (95% CI)	*P* value	Odds ratio (95% CI)	*P* value
Age, year	1.020 (0.997–1.044)	0.092	–	–
Modified frailty index				
0.00	Reference		Reference	
0.09	1.173 (0.759–1.813)	0.471	1.113 (0.703–1.764)	0.648
0.18	1.629 (1.032–2.571)	0.036	1.519 (0.931–2.476)	0.094
0.27	2.305 (1.393–3.815)	0.001	2.250 (1.316–3.848)	0.003
0.36	3.413 (1.943–5.998)	< 0.001	3.663 (1.996–6.721)	< 0.001
≥ 0.45	6.333 (2.921–13.73)	< 0.001	5.495 (2.396–12.60)	< 0.001
Malnutrition ^b^	2.041 (1.476–2.822)	< 0.001	1.522 (1.068–2.170)	0.020
Severe arrhythmia ^c^	1.638 (1.008–2.661)	0.046	–	–
Obstructive sleep apnea ^d^	3.347 (1.790–6.258)	< 0.001	2.776 (1.379–5.586)	0.004
Major neurodegenerative diseases ^e^	3.416 (1.259–9.273)	0.016	–	–
Visual/hearing impairment	1.918 (0.926–3.974)	0.080	–	–
Chronic renal insufficiency ^f^	2.199 (1.068–4.530)	0.033	–	–
Chronic hepatic dysfunction ^g^	2.216 (1.237–3.967)	0.007	–	–
Malignant tumor	2.238 (1.468–3.411)	< 0.001	–	–
Hemoglobin < 90 g/L	2.894 (1.827–4.582)	< 0.001	1.794 (1.072–3.001)	0.026
Albumin ≤30 g/L	3.327 (1.804–6.136)	< 0.001	2.051 (1.032–4.078)	0.040
Surgery by Operative Stress Score ^h^				
Low stress	Reference		Reference	
Moderate stress	13.05 (1.764–96.51)	0.012	10.34 (1.371–78.04)	0.023
High stress	26.04 (3.573–189.7)	0.001	15.86 (2.106–119.4)	0.007
Very high stress	50.77 (6.675–386.1)	< 0.001	22.40 (2.755–182.2)	0.004
Duration of surgery, hour	1.312 (1.200–1.434)	< 0.001	1.127 (1.003–1.266)	0.045
Estimated blood loss, 100 ml	1.094 (1.045–1.146)	< 0.001	-	-

^a^ Factors with *P* values < 0.20 in univariate analyses or considered clinically important were included in the model. Body mass index was excluded because it was covered by malnutrition; ASA classification was not included because of correlation with the modified frailty index; type of surgery was not included because of correlation with the surgery by Operative Stress Score; intraoperative blood transfusion was not included due to correlation with preoperative anemia or estimated blood loss. The multivariable logistic regression analysis was performed with the backward stepwise method. Hosmer-Lemeshow test for goodness of fit of the multivariable model: *χ*^2^ = 11.657, *df* = 8, *P =* 0.167

^b^ Defined by any of the following: (1) a body mass index of less than 18.5 kg/m^2^; (2) unintentional weight loss of greater than 10% within the last 3–6 months; (3) a body mass index of less than 20 kg/m^2^ and unintentional weight loss of greater than 5% within the last 3–6 months [33]

^c^ Include atrial fibrillation, frequent (> 6 beats/min) or multifocal ventricular premature beat, paroxysmal supraventricular tachycardia, second/third-degree atrioventricular block, and sick sinus syndrome

^d^ Diagnosed by previous polysomnography, or history inquiry and physical examination, and/or STOP-Bang/Berlin questionnaire

^e^ Include Alzheimer’s disease, Parkinson’s disease, and dementia

^f^ Refers to estimated glomerular filtration rate < 45 ml/min/1.73 m^2^ or on dialysis [37]. The CKD-EPI equation was adopted to calculate the estimated glomerular filtration rate [38]

^g^ Defined as Child-Pugh class B and C

^h^ Stratified into five categories of physiologic stress, i.e., very low stress, low stress, moderate stress, high stress, and very high stress [34]. Also see Supplementary Table 2

### Association between composite risk index and POCs

According to the above multivariable analysis results, a cutoff point of 0.27 was adopted to dichotomously divide patients according to the mFI. Four independent factors which represent various aspects of frailty, i.e., mFI of ≥0.27, malnutrition, hemoglobin < 90 g/L, and albumin ≤30 g/L, were combined to generate a composite risk index, each assigned with 1 point. As the composite risk index increased from 0 to 3 or above, the incidence of POCs in the four subgroups increased accordingly (Fig. 2B). Univariable analysis revealed that high composite risk index was associated with an increased risk of POCs (Supplementary Table 6).

Twelve factors, including the composite risk index, were included in a multivariable regression model. After correction for confounding factors, the composite risk index remained to be significantly associated with an increased risk of POCs; when compared with patients with a composite risk index of 0, the ORs were 2.408 (95% CI 1.714–3.383, *P* <  0.001) for those with a composite risk index of 1, 3.235 (95% CI 1.985–5.272, *P* <  0.001) for those with a composite risk index of 2, and 9.227 (95% CI 3.568–23.86, *P* <  0.001) for those with a composite risk index of 3 or above. Among other independent factors, obstructive sleep apnea (OR 2.817, 95% CI 1.400–5.670, *P* = 0.004), surgery with moderate or higher stress (compared with low-stress procedures, moderate-stress procedures: OR 10.23, 95% CI 1.347–77.71, *P* = 0.025; high-stress procedures: OR 15.55, 95% CI 2.052–117.9, *P* = 0.008; very high-stress procedures: OR 22.82, 95% CI 2.791–186.7, *P* = 0.004) were also associated with increased risk of POCs (Table 3).

**Table 3:** Effects of preoperative composite risk index in predicting postoperative complications

Variables	Univariable analyses		Multivariable analysis ^a^	
	Odds ratio (95% CI)	*P* value	Odds ratio (95% CI)	*P* value
Age, year	1.020 (0.997–1.044)	0.092	–	–
Composite risk index ^b^				
0	Reference		Reference	
1	2.604 (1.878–3.612)	< 0.001	2.408 (1.714–3.383)	< 0.001
2	3.898 (2.437–6.236)	< 0.001	3.235 (1.985–5.272)	< 0.001
≥ 3	10.61 (4.239–26.54)	< 0.001	9.227 (3.568–23.86)	< 0.001
Severe arrhythmia ^c^	1.638 (1.008–2.661)	0.046	–	–
Obstructive sleep apnea ^d^	3.347 (1.790–6.258)	< 0.001	2.817 (1.400–5.670)	0.004
Major neurodegenerative diseases ^e^	3.416 (1.259–9.273)	0.016	–	–
Visual/hearing impairment	1.918 (0.926–3.974)	0.080	–	–
Chronic renal insufficiency ^f^	2.199 (1.068–4.530)	0.033	–	–
Chronic hepatic dysfunction ^g^	2.216 (1.237–3.967)	0.007	–	–
Malignant tumor	2.238 (1.468–3.411)	< 0.001	–	–
Surgery by Operative Stress Score ^h^				
Low stress	Reference		Reference	
Moderate stress	13.05 (1.764–96.51)	0.012	10.23 (1.347–77.71)	0.025
High stress	26.04 (3.573–189.7)	0.001	15.55 (2.052–117.9)	0.008
Very high stress	50.77 (6.675–386.1)	< 0.001	22.82 (2.791–186.7)	0.004
Duration of surgery, hour	1.312 (1.200–1.434)	< 0.001	–	–
Estimated blood loss, 100 ml	1.094 (1.045–1.146)	< 0.001	–	–

^a^ Factors with *P* values < 0.20 in univariate analyses or considered clinically important were included in the model. Body mass index was excluded because it was covered by malnutrition; ASA classification was not included because of correlation with the modified frailty index; type of surgery was not included because of correlation with the surgery by Operative Stress Score; intraoperative blood transfusion was not included due to correlation with preoperative anemia or estimated blood loss. The multivariable logistic regression analysis was performed with the backward stepwise method. Hosmer-Lemeshow test for goodness of fit of the multivariable model: *χ*^2^ = 5.634, *df* = 8, *P =* 0.688

^b^ A composite of four items, i.e., modified frailty index ≥0.27, malnutrition [33], moderate or severe anemia (hemoglobin < 90 g/L), and severe hypoalbuminemia (albumin ≤30 g/L). Each item was assigned the same weight of 1 point

^c^ Include atrial fibrillation, frequent (> 6 beats/min) or multifocal ventricular premature beat, paroxysmal supraventricular tachycardia, second/third-degree atrioventricular block, and sick sinus syndrome

^d^ Diagnosed by previous polysomnography, or history inquiry and physical examination, and/or STOP-Bang/Berlin questionnaire

^e^ Include Alzheimer’s disease, Parkinson’s disease and dementia

^f^ Refers to estimated glomerular filtration rate < 45 ml/min/1.73 m^2^ or on dialysis [37]. The CKD-EPI equation was adopted to calculate the estimated glomerular filtration rate [38]

^g^ Defined as Child-Pugh class B and C

^h^ Stratified into five categories of physiologic stress, i.e., very low stress, low stress, moderate stress, high stress, and very high stress [34]. Also see Supplementary Table 2

### Postoperative outcomes according to mFI and composite risk index

Compared with patients with a mFI of < 0.27, those with a mFI of ≥0.27 had a higher incidence of POCs (23.1% [161/698] vs. 42.7% [96/225], *P* <  0.001) and a higher rate of ICU admission (18.5% [129/698] vs. 47.6% [107/225], *P* <  0.001); they also had longer lengths of ICU stay (median 21.0 h [interquartile range 17.0–39.0] vs. 28.0 h [19.0–71.0], *P* = 0.018), hospital stay (16.0 days [13.0–20.0] vs. 19.0 days [15.0–26.0], *P* <  0.001), and hospital stay after surgery (9.0 days [8.0–12.0] vs. 10.0 days [8.0–13.0], *P* = 0.002; Table 4).

**Table 4:** Postoperative outcomes according to modified frailty index and composite risk index

	Modified frailty index		*P* value	Composite risk index ^a^			*P* value
	< 0.27 (*n* = 698)	≥0.27 (*n* = 225)		0 (*n* = 502)	1 (*n* = 306)	≥2 (*n* = 115)	
Postoperative complications ^b^	161 (23.1%)	96 (42.7%)	**< 0.001**	89 (17.7%)	110 (35.9%)^*^	58 (50.4%)^*†^	**< 0.001**
Clavien-Dindo classification ^c^							
Grade III or higher complications	57 (8.2%)	40 (17.8%)	**< 0.001**	24 (4.8%)	36 (11.8%)^*^	37 (32.2%)^*†^	**< 0.001**
Grade IV or higer complications	36 (5.2%)	28 (12.4%)	**< 0.001**	15 (3.0%)	20 (6.5%)^*^	29 (25.2%)^*†^	**< 0.001**
Grade V complications	4 (0.6%)	3 (1.3%)	0.483	2 (0.4%)	0 (0.0%)	5 (4.3%)^*†^	**< 0.001**
ICU admission	129 (18.5%)	107 (47.6%)	**< 0.001**	74 (14.7%)	101 (33.0%)^*^	61 (53.0%)^*†^	**< 0.001**
LOS in ICU, hour ^d^	21.0 (17.0, 39.0)	28.0 (19.0, 71.0)	**0.018**	20.0 (17.0, 32.5)	24.0 (18.0, 56.5)	32.0 (20.5, 96.0)^*†^	**< 0.001**
Unplanned reintubation	6 (0.9%)	6 (2.7%)	0.081	4 (0.8%)	3 (1.0%)	5 (4.3%)^*^	**0.027**
Unplanned reoperation	21 (3.0%)	7 (3.1%)	0.938	14 (2.8%)	13 (4.2%)	1 (0.9%)	0.197
Hospital LOS, day	16.0 (13.0, 20.0)	19.0 (15.0, 26.0)	**< 0.001**	16.0 (12.8, 19.0)	18.0 (15.0, 24.0) ^*^	21.0 (16.0, 27.0) ^*†^	**< 0.001**
Hospital LOS after surgery, day	9.0 (8.0, 12.0)	10.0 (8.0, 13.0)	**0.002**	9.0 (7.0, 11.0)	10.0 (8.0, 13.0)^*^	11.0 (9.0, 14.0)^*^	**< 0.001**
Adverse discharge destination ^e^	8 (1.1%)	7 (3.1%)	0.085	6 (1.2%)	6 (2.0%)	3 (2.6%)	0.399

Data are n (%) or median (interquartile range). *P* values in bold indicate < 0.05. ^*^P < 0.05/3 = 0.017 (Bonferroni-corrected post hoc multiple comparisons) when compared with the patients with a composite risk index of 0. ^†^*P* < 0.017 when compared with the patients with a composite risk index of 1

*ICU* intensive care unit, *LOS* length of stay

^a^ A composite of four items, i.e., modified frailty index ≥0.27, malnutrition [33], moderate or severe anemia (hemoglobin < 90 g/L), and severe hypoalbuminemia (albumin ≤30 g/L). Each item was assigned the same weight of 1 point

^b^ Indicate those of Clavien-Dindo grade II or higher. Also see Supplementary Table 4

^c^ Clavien-Dindo classification of postoperative complications [35]

^d^ Results of patients who were admitted to the ICU

^e^ Defined as discharge to destinations other than home (e.g., a long- or short-term care facility)

Compared with patients with a composite risk index of 0, those with a composite risk index of 1 and ≥ 2 had higher incidences of POCs (17.7% [89/502] with 0 vs. 35.9% [110/306] with 1 vs. 50.4% [58/115] with ≥2, *P* <  0.001), higher rates of ICU admission (14.7% [74/502] with 0 vs. 33.0% [101/306] with 1 vs. 53.0% [61/115] with ≥2, *P* <  0.001), and higher rate of unplanned reintubation (0.8% [4/502] with 0 vs. 1.0% [3/306] with 1 vs. 4.3% [5/115] with ≥2, *P =* 0.027); they also had longer lengths of ICU stay (median 20.0 h [interquartile range 17.0–32.5] with 0 vs. 24.0 h [18.0–56.5] with 1 vs. 32.0 h [20.5–96.0] with ≥2, *P* <  0.001), hospital stay (16.0 days [12.8–19.0] with 0 vs. 18.0 days [15.0–24.0] with 1 vs. 21.0 days [16.0–27.0] with ≥2, *P* <  0.001), and hospital stay after surgery (9.0 days [7.0–11.0] with 0 vs. 10.0 days [8.0–13.0] with 1 vs. 11.0 days [9.0–14.0] with ≥2, *P* <  0.001; Table 4).

### Comparison of mFI and composite risk index for POCs prediction

The area under receiver-operator characteristic curve of mFI in predicting POCs was 0.622 (95% CI 0.581–0.663, *P* <  0.001); that of composite risk index in predicting POCs was 0.653 (95% CI 0.613–0.694, *P* <  0.001). There was no significant difference in the discriminative power between the two instruments (Supplementary Fig. 1).

## Discussion

Our results confirmed that high composite risk index, a combination of mFI (≥0.27), malnutrition, moderate to severe anemia (hemoglobin < 90 g/L), and severe hypoalbuminemia (albumin ≤30 g/L), was an independent predictor for increased risk of POCs in older patients recovering from elective digestive tract surgery. Furthermore, there was a “dose-effect” relationship, i.e., the higher the composite risk index, the higher the incidence of POCs.

In the present study, POCs occurred in 27.8% of older patients following elective digestive tract surgery. In previous studies of patients undergoing various digestive tract surgeries, the reported incidence of POCs varied from 26.9 to 45.2% [39–42]; the incidence of POCs in our patients was well within this range. Along with the aging population, the number of older patients undergoing surgical procedures has been increasing in recent years. However, despite improvements in perioperative management, the incidence of POCs in older patients remains higher than that in young patients [16, 24]. Therefore, it is extremely necessary to identify the risk factors of POCs in older surgical patients. As one of the age-related factors, frailty is attracting more and more attention.

The frailty index evaluates “accumulated deficits” across multiple domains involving the functional, cognitive, emotional, sleep, nutritional, social, and medical history. The score of frailty index is obtained by dividing the sum of deficits present by the total number of deficits measured [9]. The measurement process of the frailty index, however, is time-consuming and necessitates professional skills [6, 43]. As a shortened scale, the mFI consists of 10 items on comorbidities and 1 item on functional status. It can be easily acquired from routine clinical practice, either prospectively or retrospectively [7]. The effect of mFI has been validated in patients scheduled for elective digestive tract surgery. In a retrospective study of 58,448 adult patients undergoing colectomies, Obeid et al. [40] found a significant association between mFI and POCs; the incidence of serious POCs (Clavien-Dindo class IV/V) increased from 3.2 to 56.3% as the mFI score increased from 0 to 0.55. In another retrospective study of 9986 adult patients undergoing pancreaticoduodenectomy, Mogal et al. [41] reported that high mFI (≥0.27) was significantly associated with increased risks of any complications, major complications (Clavien-Dindo class III or higher), and 30-day mortality. Consistent with previous studies [16–24, 40, 41], high mFI score was also independently associated with an increased risk of POCs in our patients. Specifically, we found that those with a mFI of ≥0.27 developed more POCs; they also required more ICU admission, and stayed longer in the ICU and the hospital. We therefore adopted ≥0.27 as the cut-off point of the mFI. Similar cut-off point was also suggested by some others [21, 41].

It should be noted that the mFI does not fully evaluate the entire spectrum of frailty because it consists of only two domains (comorbidities and functional decline). In our results, other frailty-related parameters, including malnutrition, moderate to severe anemia (hemoglobin < 90 g/L), and severe hypoalbuminemia (albumin ≤30 g/L), were also independently associated with increased risk of POCs. As an important dimension of frailty [6], malnutrition is common among older surgical patients and is related to increased perioperative morbidity and other worse outcomes [25, 44, 45]. Considering the data availability in our medical records system, we defined malnutrition using the NICE criteria which focuses on weight loss and body mass index [33]. The rate of malnutrition was 23.0% in our patients, like other studies in a similar patient population [45, 46]. The prevalence of anemia increases with age [47, 48], mainly due to nutrient deficiency, chronic renal disease and/or inflammation, and unexplained reasons [49]. Although controversial, serum albumin is still recommended for preoperative nutritional screening [50]. Hypoalbuminemia is a valid predictor of poor postoperative outcomes [26, 27, 51]. In the present study, we enrolled patients undergoing digestive system surgeries; the majority (80.3%) of them turned out to have digestive tract malignancies. Our patients were at high risk of malnutrition, anemia, and hypoalbuminemia.

Since the above four risk factors are all related to frailty characteristics of the study patients and are easily acquired in routine clinical practice, it is feasible to use the combination of these factors as an evaluation tool of preoperative frailty. We therefore tested the value of a composite risk index, a combination of mFI ≥0.27, malnutrition, moderate to severe anemia, and severe hypoalbuminemia, in predicting the risk of POCs in our patients. Our results showed that patients with a high composite risk index developed more POCs, required more ICU admission and unplanned reintubation, and stayed longer in the ICU and the hospital. Multivariable analysis also confirmed that higher composite risk index was associated with higher risk of POCs. The effect of the composite risk index in predicting POCs is similar, if not superior, to that of the mFI. Our results may help perioperative clinicians to better predict the postoperative outcomes and help patients for decision-making before surgery. Furthermore, since the three parameters added to mFI in the composite risk index are all modifiable, our results indicate potential targets of intervention. Further studies are required to explore whether preoperative individualized intervention can improve outcomes of these high-risk patients.

Our study had several limitations. First, the study was performed retrospectively with data not specifically intended for frailty assessment; data on other frailty domains such as sarcopenia, and psychosocial and cognitive parameters, were unavailable. These might lead to an underestimation of the frailty syndrome. Second, the primary outcome of our study was limited to in-hospital POCs; the occurrence of post-discharge complications was not collected. These may confound the effects of frailty on the outcomes. Finally, as a single institution study, our results may not be extrapolated to patients in other centers. Despite these, our findings have clinical significance for improvement of perioperative care and management and generate hypotheses for further exploration.

## Conclusions

Our results showed that high preoperative composite risk index, a combination of frailty (mFI ≥0.27), malnutrition, moderate to severe anemia (hemoglobin < 90 g/L), and severe hypoalbuminemia (albumin ≤30 g/L), was independently associated with an increased risk of in-hospital POCs in older patients undergoing elective digestive tract surgery. Further studies are required to explore whether individualized preoperative intervention can improve outcomes in high-risk patients.

## Supplementary Information

**Additional file 1: Supplementary Table 1** Composition of Modified Frailty Index.

**Additional file 2: Supplementary Table 2** Surgical procedures stratified according to Operative Stress Score.

**Additional file 3: Supplementary Table 3** Clavien**-**Dindo classification of postoperative complications.

**Additional file 4: Supplementary Table 4** Individual complications of Clavien-Dindo classification grade II or higher.

**Additional file 5: Supplementary Table 5** Baseline and intraoperative data according to modified frailty index and composite risk index.

**Additional file 6: Supplementary Table 6** Factors in association with postoperative complications (univariate analyses).

**Additional file 7: Supplementary Figure 1** The area under receiver-operator characteristic curves of modified frailty index and composite risk index in predicting postoperative complications.

**Additional file 8:** STROBE Statement—Checklist of items that should be included in reports of cohort studies.

## Data Availability

The datasets that support the findings of the study are available from the corresponding author on reasonable request.

## Abbreviations

MFI: Modified Frailty Index
POCs: Postoperative complications
ASA: American Society of Anesthesiologists
ICU: Intensive care unit
ROC: Receiver-operating characteristic
PNB: Peripheral nerve block
LOS: Length of stay
OR: Odds ratio
CI: Confidential interval

## References

[1] Clegg A, Young J, Iliffe S, Rikkert MO, Rockwood K (2013). Frailty in elderly people. Lancet..

[2] Makary MA, Segev DL, Pronovost PJ, Syin D, Bandeen-Roche K, Patel P (2010). Frailty as a predictor of surgical outcomes in older patients. J Am Coll Surg.

[3] Fang EF, Scheibye-Knudsen M, Jahn HJ, Li J, Ling L, Guo H (2015). A research agenda for aging in China in the 21st century. Ageing Res Rev.

[4] Kwok AC, Semel ME, Lipsitz SR, Bader AM, Barnato AE, Gawande AA (2011). The intensity and variation of surgical care at the end of life: a retrospective cohort study. Lancet..

[5] Griffiths R, Beech F, Brown A, Dhesi J, Foo I, Goodall J (2014). Perioperative care of the elderly 2014: Association of Anaesthetists of Great Britain and Ireland. Anaesthesia..

[6] Alvarez-Nebreda ML, Bentov N, Urman RD, Setia S, Huang JC, Pfeifer K (2018). Recommendations for preoperative management of frailty from the Society for Perioperative Assessment and Quality Improvement (SPAQI). J Clin Anesth.

[7] Velanovich V, Antoine H, Swartz A, Peters D, Rubinfeld I (2013). Accumulating deficits model of frailty and postoperative mortality and morbidity: its application to a national database. J Surg Res.

[8] Fried LP, Tangen CM, Walston J, Newman AB, Hirsch C, Gottdiener J (2001). Frailty in older adults: evidence for a phenotype. J Gerontol A Biol Sci Med Sci.

[9] Mitnitski AB, Mogilner AJ, Rockwood K (2001). Accumulation of deficits as a proxy measure of aging. Sci World J.

[10] Hall DE, Arya S, Schmid KK, Blaser C, Carlson MA, Bailey TL (2017). Development and initial validation of the risk analysis index for measuring frailty in surgical populations. JAMA Surg.

[11] Rolfson DB, Majumdar SR, Tsuyuki RT, Tahir A, Rockwood K (2006). Validity and reliability of the Edmonton frail scale. Age Ageing.

[12] Morley JE, Malmstrom TK, Miller DK (2012). A simple frailty questionnaire (FRAIL) predicts outcomes in middle aged African Americans. J Nutr Health Aging.

[13] Peters LL, Boter H, Buskens E, Slaets JP (2012). Measurement properties of the Groningen frailty indicator in home-dwelling and institutionalized elderly people. J Am Med Dir Assoc.

[14] Wang SL, Zhuang CL, Huang DD, Pang WY, Lou N, Chen EF (2016). Sarcopenia adversely impacts postoperative clinical outcomes following gastrectomy in patients with gastric cancer: a prospective study. Ann Surg Oncol.

[15] Rockwood K, Song X, MacKnight C, Bergman H, Hogan DB, McDowell I (2005). A global clinical measure of fitness and frailty in elderly people. CMAJ..

[16] Seib CD, Rochefort H, Chomsky-Higgins K, Gosnell JE, Suh I, Shen WT (2018). Association of patient frailty with increased morbidity after common ambulatory general surgery operations. JAMA Surg.

[17] Louwers L, Schnickel G, Rubinfeld I (2016). Use of a simplified frailty index to predict Clavien 4 complications and mortality after hepatectomy: analysis of the National Surgical Quality Improvement Project database. Am J Surg.

[18] Farhat JS, Velanovich V, Falvo AJ, Horst HM, Swartz A, Patton JH (2012). Are the frail destined to fail? Frailty index as predictor of surgical morbidity and mortality in the elderly. J Trauma Acute Care Surg.

[19] George EM, Burke WM, Hou JY, Tergas AI, Chen L, Neugut AI (2016). Measurement and validation of frailty as a predictor of outcomes in women undergoing major gynaecological surgery. Bjog..

[20] Shin JI, Keswani A, Lovy AJ, Moucha CS (2016). Simplified frailty index as a predictor of adverse outcomes in total hip and knee arthroplasty. J Arthroplast.

[21] Kessler RA, De la Garza RR, Purvis TE, Ahmed AK, Goodwin CR, Sciubba DM (2018). Impact of frailty on complications in patients with thoracic and thoracolumbar spinal fracture. Clin Neurol Neurosurg.

[22] Abt NB, Richmon JD, Koch WM, Eisele DW, Agrawal N (2016). Assessment of the predictive value of the modified frailty index for Clavien-Dindo grade IV critical care complications in major head and neck cancer operations. JAMA Otolaryngol Head Neck Surg.

[23] Murphy PB, Savage SA, Zarzaur BL (2020). Impact of patient frailty on morbidity and mortality after common emergency general surgery operations. J Surg Res.

[24] Youngerman BE, Neugut AI, Yang J, Hershman DL, Wright JD, Bruce JN (2018). The modified frailty index and 30-day adverse events in oncologic neurosurgery. J Neuro-Oncol.

[25] Nakagawa T, Toyazaki T, Chiba N, Ueda Y, Gotoh M (2016). Prognostic value of body mass index and change in body weight in postoperative outcomes of lung cancer surgery. Interact Cardiovasc Thorac Surg.

[26] Hendifar A, Osipov A, Khanuja J, Nissen N, Naziri J, Yang W (2016). Influence of body mass index and albumin on perioperative morbidity and clinical outcomes in resected pancreatic adenocarcinoma. PLoS One.

[27] Haskins IN, Baginsky M, Amdur RL, Agarwal S (2017). Preoperative hypoalbuminemia is associated with worse outcomes in colon cancer patients. Clin Nutr.

[28] Xia L, Guzzo TJ (2017). Preoperative anemia and low hemoglobin level are associated with worse clinical outcomes in patients with bladder cancer undergoing radical cystectomy: a meta-analysis. Clin Genitourin Cancer.

[29] D'Cruz RT, Chong TT, Tan TF, Ting ZYP, Lee QWS, Wong TH (2020). The modified frailty index does not predict mortality after major lower extremity amputation for peripheral arterial disease in an asian population. Ann Vasc Surg.

[30] Elsamadicy AA, Freedman IG, Koo AB, David WB, Reeves BC, Havlik J (2021). Modified-frailty index does not independently predict complications, hospital length of stay or 30-day readmission rates following posterior lumbar decompression and fusion for spondylolisthesis. Spine J.

[31] Mohd Rothi I, Deverall HH, Baker JF (2019). The modified frailty index does not correlate with survival in surgically-treated patients with metastatic spine disease. J Clin Neurosci.

[32] Li YM, Fan JG (2019). Guidelines of prevention and treatment for alcoholic liver disease (2018, China). J Dig Dis.

[33] National Collaborating Centre for Acute Care (UK) (2006). Nutrition support for adults: oral nutrition support, enteral tube feeding and parenteral nutrition [R].

[34] Shinall MC, Arya S, Youk A, Varly P, Shah R, Massarweh NN (2019). Association of preoperative patient frailty and operative stress with postoperative mortality. JAMA Surg.

[35] Dindo D, Demartines N, Clavien PA (2004). Classification of surgical complications: a new proposal with evaluation in a cohort of 6336 patients and results of a survey. Ann Surg.

[36] Peduzzi P, Concato J, Kemper E, Holford TR, Feinstein AR (1996). A simulation study of the number of events per variable in logistic regression analysis. J Clin Epidemiol.

[37] Levin A, Stevens PE (2014). Summary of KDIGO 2012 CKD guideline: behind the scenes, need for guidance, and a framework for moving forward. Kidney Int.

[38] Levey AS, Stevens LA, Schmid CH, Zhang YL, Castro AF, Feldman HI (2009). A new equation to estimate glomerular filtration rate. Ann Intern Med.

[39] Chen SY, Stem M, Cerullo M, Gearhart SL, Safar B, Fang SH (2018). The effect of frailty index on early outcomes after combined colorectal and liver resections. J Gastrointest Surg.

[40] Obeid NM, Azuh O, Reddy S, Webb S, Reickert C, Velanovich V (2012). Predictors of critical care-related complications in colectomy patients using the National Surgical Quality Improvement Program: exploring frailty and aggressive laparoscopic approaches. J Trauma Acute Care Surg.

[41] Mogal H, Vermilion SA, Dodson R, Hsu FC, Howerton R, Shen P (2017). Modified frailty index predicts morbidity and mortality after pancreaticoduodenectomy. Ann Surg Oncol.

[42] Tanaka R, Lee SW, Kawai M, Tashiro K, Kawashima S, Kagota S (2017). Protocol for enhanced recovery after surgery improves short-term outcomes for patients with gastric cancer: a randomized clinical trial. Gastric Cancer.

[43] Cesari M, Gambassi G, van Kan GA, Vellas B (2014). The frailty phenotype and the frailty index: different instruments for different purposes. Age Ageing.

[44] Thomas MN, Kufeldt J, Kisser U, Hornung HM, Hoffmann J, Andraschko M (2016). Effects of malnutrition on complication rates, length of hospital stay, and revenue in elective surgical patients in the G-DRG-system. Nutrition..

[45] Kim E, Lee DH, Jang JY (2019). Effects of preoperative malnutrition on postoperative surgical outcomes and quality of life of elderly patients with periampullary neoplasms: a single-center prospective cohort study. Gut Liver.

[46] Zheng HL, Lu J, Li P, Xie JW, Wang JB, Lin JX (2017). Effects of preoperative malnutrition on short- and long-term outcomes of patients with gastric cancer: can we do better?. Ann Surg Oncol.

[47] Denny SD, Kuchibhatla MN, Cohen HJ (2006). Impact of anemia on mortality, cognition, and function in community-dwelling elderly. Am J Med.

[48] Phillips R, Wood H, Weaving G, Chevassut T (2021). Changes in full blood count parameters with age and sex: results of a survey of almost 900 000 patient samples from primary care. Br J Haematol.

[49] Guralnik JM, Eisenstaedt RS, Ferrucci L, Woodman RC (2004). Prevalence of anemia in persons 65 years and older in the United States: evidence for a high rate of unexplained anemia. Blood..

[50] McClave SA, Kozar R, Martindale RG, Heyland DK, Braga M, Carli F (2013). Summary points and consensus recommendations from the north American surgical nutrition summit. JPEN J Parenter Enteral Nutr.

[51] Zhang DF, Su X, Meng ZT, Cui F, Li HL, Wang DX (2018). Preoperative severe hypoalbuminemia is associated with an increased risk of postoperative delirium in elderly patients: results of a secondary analysis. J Crit Care.

