# Supplementary material for: The composite risk index based on frailty predicts postoperative complications in older patients recovering from elective digestive tract surgery: a retrospective cohort study
Source: BMC Anesthesiol. 2022 Jan 3;22:7. doi: 10.1186/s12871-021-01549-6 (PMC8722296; doi:10.1186/s12871-021-01549-6)
Supplement: Supplementary file 1 — Additional file 1: Supplementary Table 1 Composition of Modified Frailty Index. [file 12871_2021_1549_MOESM1_ESM.docx]

**Supplement Table 1** Composition of Modified Frailty Index [7]

|  | All patients | Without postoperative complications | With postoperative complications | *P* value |
| --- | --- | --- | --- | --- |
|  | (n = 923) | (n = 666) | (n = 257) |  |
| Hypertension with medication | 467 (50.6%) | 339 (50.9%) | 128 (49.8%) | 0.765 |
| Diabetes mellitus | 233 (25.2%) | 139 (20.9%) | 94 (36.6%) | **<0.001** |
| COPD or current pneumonia | 91 (9.9%) | 56 (8.4%) | 35 (13.6%) | **0.017** |
| CHF exacerbation within 30 days | 12 (1.3%) | 4 (0.6%) | 8 (3.1%) | **0.007** |
| MI within 6 months | 5 (0.5%) | 1 (0.2%) | 4 (1.6%) | **0.035** |
| Angina within 1 month or PCI/CABG | 176 (19.1%) | 105 (15.8%) | 71 (27.6%) | **<0.001** |
| TIA/stroke without deficits | 166 (18.0%) | 111 (16.7%) | 55 (21.4%) | 0.093 |
| Stroke with deficits | 37 (4.0%) | 18 (2.7%) | 19 (7.4%) | **0.001** |
| Peripheral vascular disease | 105 (11.4%) | 59 (8.9%) | 46 (17.9%) | **<0.001** |
| Acutely impaired sensorium ^a^ | 2 (0.2%) | 0 (0%) | 2 (0.8%) | 0.077 |
| Partially or totally dependent | 207 (22.4%) | 126 (18.9%) | 81 (31.5%) | **<0.001** |
| Daily functional status |  |  |  | **<0.001** |
| Independent | 716 (77.6%) | 540 (81.1%) | 176 (68.5%) |  |
| Partially dependent | 198 (21.5%) | 124 (18.6%) | 74 (28.8%) |  |
| Totally dependent | 9 (1.0%) | 2 (0.3%) | 7 (2.7%) |  |

Data are n (%). *P* values in bold indicate <0.05.

*COPD* chronic obstructive pulmonary disease, *CHF* congestive heart failure*, MI* myocardial infarction, *PCI* percutaneous coronary intervention, *CABG* coronary artery bypass surgery, *TIA* transient ischemic attack

^a^ Refers to acute mental status changes and/or delirium in the context of the current illness. Patients with chronic or long-standing mental status changes secondary to chronic mental illness or chronic dementing illnesses are not included.
