# Supplementary material for: The composite risk index based on frailty predicts postoperative complications in older patients recovering from elective digestive tract surgery: a retrospective cohort study
Source: BMC Anesthesiol. 2022 Jan 3;22:7. doi: 10.1186/s12871-021-01549-6 (PMC8722296; doi:10.1186/s12871-021-01549-6)
Supplement: Supplementary file 2 — Additional file 2: Supplementary Table 2 Surgical procedures stratified according to Operative Stress Score. [file 12871_2021_1549_MOESM2_ESM.docx]

**Supplementary Table 2** Surgical procedures stratified according to Operative Stress Score [34]

| Category, Procedure Type | All (n = 923) |
| --- | --- |
| **Category 1, Very Low Stress** |  |
| All | 0 (0.0%) |
| **Category 2, Low Stress** |  |
| All | 56 (6.1%) |
| Appendectomy, unruptured, laparoscopic or open | 32 (3.5%) |
| Inguinal hernia, laparoscopic or open | 24 (2.6%) |
| **Category 3, Moderate Stress** |  |
| All | 266 (28.8%) |
| Cholecystectomy, laparoscopic or open with or without intraoperative cholangiogram | 20 (2.2%) |
| Hepatic cyst fenestration, laparoscopic | 6 (0.7%) |
| Gastric restrictive procedure, longitudinal gastrectomy, laparoscopic | 6 (0.7%) |
| Gastrostomy, laparoscopic | 7 (0.8%) |
| Closure of enterostomy, large or small intestine | 25 (2.7%) |
| Colectomy, laparoscopic | 167 (18.1%) |
| Enterectomy, enteroenterostomy | 22 (2.4%) |
| Enterolysis | 13 (1.4%) |
| **Category 4, High Stress** |  |
| All | 526 (57.0%) |
| Colectomy, open | 65 (7.0%) |
| Gastrectomy | 141 (15.3%) |
| Hepatectomy, resection of liver; partial lobectomy | 43 (4.7%) |
| Proctectomy, laparoscopic or open | 264 (28.6%) |
| Pancreatectomy, distal subtotal, with or without splenectomy; without pancreatojejunostomy | 13 (1.4%) |
| **Category 5, Very High Stress** | 75 (8.1%) |
| All | 75 (8.1%) |
| Pancreaticoduodenectomy (Whipple-type procedure) | 75 (8.1%) |

Data are n (%).
