# Supplementary material for: The composite risk index based on frailty predicts postoperative complications in older patients recovering from elective digestive tract surgery: a retrospective cohort study
Source: BMC Anesthesiol. 2022 Jan 3;22:7. doi: 10.1186/s12871-021-01549-6 (PMC8722296; doi:10.1186/s12871-021-01549-6)
Supplement: Supplementary file 3 — Additional file 3: Supplementary Table 3 Clavien-Dindo classification of postoperative complications. [file 12871_2021_1549_MOESM3_ESM.docx]

**Supplementary Table 3** Clavien**-**Dindo classification of postoperative complications [35]

| Grade | Definition | All (n = 923) |
| --- | --- | --- |
| Grade I | Any deviation from the normal postoperative course without the need for special interventions; complications that are managed with routine medication (such as analgesics, antiemetics, antipyretics, diuretics, and electrolytes) or physiotherapy, and wound infections that are managed at the bedside are classified as Grade I. | --- |
| Grade II | Requiring total parenteral nutrition, blood transfusions, or pharmacological treatment with drugs other than those allowed for Grade I complications. | 160 (17.3%) |
| Grade III | Requiring surgical, endoscopic, or radiological intervention. | 33 (3.6%) |
| Grade IV | Life-threatening complications requiring intermediate care/intensive care unit (ICU) management excluding transient ischemic attacks. | 57 (6.2%) |
| Grade V | Death of a patient. | 7 (0.8%) |

Data are n (%).
