# Supplementary material for: The composite risk index based on frailty predicts postoperative complications in older patients recovering from elective digestive tract surgery: a retrospective cohort study
Source: BMC Anesthesiol. 2022 Jan 3;22:7. doi: 10.1186/s12871-021-01549-6 (PMC8722296; doi:10.1186/s12871-021-01549-6)
Supplement: Supplementary file 4 — Additional file 4: Supplementary Table 4 Individual complications of Clavien-Dindo classification grade II or higher. [file 12871_2021_1549_MOESM4_ESM.docx]

**Supplementary Table 4** Individual complications of Clavien-Dindo classification grade II or higher

| Complications | All patients (n = 923) |
| --- | --- |
| Cardiovascular complications, n (%) | 67 (7.3%) |
| Acute coronary syndrome ^a^ | 13 (1.4%) |
| New-onset arrhythmia ^b^ | 20 (2.2%) |
| Circulatory insufficiency ^c^ | 24 (2.6%) |
| Acute heart failure or exacerbation of congestive heart failure ^d^ | 19 (2.1%) |
| Cardiac arrest ^e^ | 3 (0.3%) |
| Respiratory complications, n (%) | 71 (7.7%) |
| Respiratory infection ^f^ | 20 (2.2%) |
| Respiratory failure ^g^ | 21 (2.3%) |
| Atelectasis ^h^ | 10 (1.1%) |
| Pleural effusion ^i^ | 21 (2.3%) |
| Bronchospasm ^j^/asthma attack ^k^ | 4 (0.4%) |
| Influenza ^l^ | 2 (0.2%) |
| Aspiration pneumonia ^m^ | 1 (0.1%) |
| Neurological complications, n (%) | 23 (2.5%) |
| Stroke ^n^ | 5 (0.5%) |
| Transient ischemic attack ^o^ | 6 (0.7%) |
| Delirium ^p^ | 7 (0.8%) |
| Pulmonary encephalopathy ^q^ | 5 (0.5%) |
| Renal complications, n (%) | 24 (2.6%) |
| Acute renal failure ^r^ | 6 (0.7%) |
| Acute kidney injury ^s^ | 30 (3.3%) |
| Hepatic complications, n (%) | 16 (1.7%) |
| Acute hepatic injury ^t^ | 16 (1.7%) |
| Hematologic complications, n (%) | 37 (4.0%) |
| Venous thrombosis ^u^ | 22 (2.4%) |
| Pulmonary embolism ^v^ | 2 (0.2%) |
| Disseminated intravascular coagulation ^w^ | 2 (0.2%) |
| Blood coagulation disorder ^x^ | 13 (1.4%) |
| Surgical complications, n (%) | 133 (14.4%) |
| Wound infection ^y^ | 8 (0.9%) |
| Intra-abdominal/pelvic infection ^z^ | 30 (3.3%) |
| Wound dehiscence ^aa^ | 1 (0.1%) |
| Anastomotic fistula ^bb^ | 27 (2.9%) |
| Pancreatic fistula ^cc^ | 5 (0.5%) |
| Anastomotic stenosis ^dd^ | 1 (0.1%) |
| Mechanical bowel obstruction ^ee^ | 14 (1.5%) |
| Delayed gastrointestinal recovery ^ff^ | 54 (5.9%) |
| Surgical hemorrhage ^gg^ | 48 (5.2%) |
| Gastrointestinal hemorrhage ^hh^ | 7 (0.8%) |
| Infectious complications, n (%) | 26 (2.8%) |
| Sepsis ^ii^ | 25 (2.7%) |
| Urinary tract infection ^jj^ | 4 (0.4%) |
| Other complications | 6 (0.7%) |
| Anaphylactic reaction to antibiotics ^kk^ | 3 (0.3%) |
| Postoperative hoarseness ^ll^ | 3 (0.3%) |

Data are n (%).

^a^ Include acute myocardial infarction and unstable angina, which were confirmed by clinical symptoms, electrocardiographic changes, imaging evidences, and serum cardiac troponin I concentration.

^b^ Confirmed by 12-lead electrocardiogram and necessitated medical treatment and/or cardioversion.

^c^ Requirement of inotropics and/or vasopressors for more than 24 hours after surgery.

^d^ Diagnosed by the presence of new-onset orthopnea with evidence of fluid retention (i.e., elevated jugular venous pressure, evidence of pulmonary oedema, and/or peripheral oedema) and an elevated plasma brain natriuretic peptide of above 400 pg/mL

^e^ The absence of large artery pulsation and heart sound, subsequent loss of consciousness, respiratory arrest, dilated pupils and even death, caused by the sudden stop of heart beating.

^f^ Receiving antibiotics for suspected respiratory infection and meet at least one of the following criteria: new or changed sputum, new or changed lung opacities, fever, leukocyte count >12×10^9^/L.

^g^ PaO_2_ <60 mmHg on room air, a ratio of PaO_2_ to inspired oxygen fraction <300, or arterial oxyhemoglobin saturation measured with pulse oximetry <90% and requiring oxygen therapy.

^h^ Lung opacification with a shift of the mediastinum, hilum, or hemidiaphragm toward the affected area, and compensatory overinflation in the adjacent non-atelectatic lung.

^i^ Chest X-ray demonstrating blunting of the costophrenic angle, loss of the sharp silhouette of the ipsilateral hemidiaphragm in the upright position, evidence of displacement of adjacent anatomical structures, or (in supine position) a hazy opacity in one hemithorax with preserved vascular shadows.

^j^ Confirmed by new-onset expiratory wheezing and necessitated treatment with bronchodilators.

^k^ Diagnosed according to clinical signs and required inhaled bronchodilator therapy.

^l^ Diagnosed by detection of viral nucleic acid in respiratory samples and symptoms of influenza, including fever and muscle soreness; and necessitated antiviral therapy.

^m^ Pneumonia caused by aspiration and reflux of gastric contents

^n^ Persisted new focal neurologic deficit and confirmed by neurologic imaging.

^o^ A sudden onset of focal neurologic signs and/or symptoms that lasted for less than 24 hours. No acute infarction was found in the cranial imaging examination.

^p^ Diagnosed by psychiatric symptoms of inattention and thinking disorder, or inattention and altered level of consciousness, with an acute onset or a fluctuation course.

^q^ Neuropsychiatric syndrome caused by carbon dioxide accumulation and hypoxia due to respiratory failure, and ruled out other causes of neuropsychiatric disorders.

^r^ New onset renal failure that required renal replacement therapy.

^s^ Increase in serum creatinine by ≥0.3 mg/dl (≥26.5 μmol/l) within 48 hours, or to ≥1.5 times baseline within 7 days, or urine volume <0.5 ml/kg/h for at least 6 hours.

^t^ New-onset increase of total serum bilirubin ≥33 mol/l, requiring medical interventions such as liver-protective drugs.

^u^ Non-compressibility of one or more venous segments on B-mode ultrasonography.

^v^ Hypotension or shock suspected of pulmonary embolism, and meet one of the following: filling defect in any branch of the pulmonary artery in computed tomographic pulmonary angiogram or right ventricular overload in echocardiogram. For suspected pulmonary embolism without symptoms, positive finding in computed tomographic pulmonary angiogram was required for diagnosis.

^w^ Diagnosed by abnormal bleeding symptoms and more than three anomalies in the following items: platelet <100×10^9^/L or progressive decline; fibrinogen <1.5 g/L or progressive decline or >4 g/L; plasma fibrin degradation product (FDP) >20 mg/L or D-dimer level increased or positive, or 3P test (plasma protamine paracoagulation test) positive; prothrombin time (PT) shorter or longer than 3 s or activated partial thromboplastin time (APTT) shorter or longer than 10 s.

^x^ Diagnosed by any of the following items: platelet < 80×10^9^/L or progressive decline; prothrombin time shorter or longer than 4 s; activated partial thromboplastin time shorter or longer than 10 s; fibrinogen <1.5 g/L or progressive decline or >4 g/L.

^y^ Pus expressed from the superficial or deep incision, and bacteria cultured from the pus.

^z^ Diagnosed according to the significantly increased white blood cell count, clinic presentations of high fever, abdominal pain/distension, and peritoneal/pelvic effusion confirmed by imaging examination; or the purulent fluid extracted by puncture and bacteria cultured from the fluid.

^aa^ Wound rupture that required secondary suturing or antibiotics.

^bb^ Extravasation of contrast agent in the body cavity or retroperitoneal space during imaging examination, or anastomosis-associated abdominal pain and peritonitis with changes in laboratory indicators, or perianastomotic abscess.

^cc^ Increase of amylase level in drainage fluid ≥3 times the upper limit of institutional criteria, on or after postoperative day 3.

^dd^ Diagnosis supported by imaging examination or endoscopy, and excluded the presence of gastrointestinal dysfunction and mechanical obstruction.

^ee^ Lack of bowel movement, flatulence, and requirement of intravenous fluid therapy for more than one week after surgery; confirmed by imaging examination or secondary surgery.

^ff^ Manifested by the symptoms of paralytic ileus or delayed gastric emptying, including nausea and vomiting, abdominal distension and discomfort, absence of passage of flatus or stool, accumulation of gas and fluid in the bowel, intolerance to oral intake, and ruled out stenosis and mechanical obstruction.

^gg^ Bleeding after surgery that required blood transfusion or secondary surgical hemostasis

^hh^ Hematemesis, black stool, changes in drainage/hemoglobin, and other direct or indirect evidence suggests the possibility of bleeding.

^ii^ Two or more criteria of systemic inflammatory response syndrome, with known infection and new-onset dysfunction of at least one organ/system.

^jj^ Confirmed by urinalysis and urine culture and necessitated antibiotic therapy.

^kk^ Acute onset of severe allergic reaction after exposure to antibiotics and necessitated intervention.

^ll^ Postoperative hoarseness requiring treatment with nebulization or drug.
