# Supplementary material for: The composite risk index based on frailty predicts postoperative complications in older patients recovering from elective digestive tract surgery: a retrospective cohort study
Source: BMC Anesthesiol. 2022 Jan 3;22:7. doi: 10.1186/s12871-021-01549-6 (PMC8722296; doi:10.1186/s12871-021-01549-6)
Supplement: Supplementary file 5 — Additional file 5: Supplementary Table 5 Baseline and intraoperative data according to modified frailty index and composite risk index. [file 12871_2021_1549_MOESM5_ESM.docx]

**Supplementary Table 5** Baseline and intraoperative data according to modified frailty index and composite risk index

|  | Modified frailty index | | *P* value | Composite risk index | | | *P* value |
| --- | --- | --- | --- | --- | --- | --- | --- |
|  | <0.27 (n = 698) | ≥0.27 (n = 225) |  | 0 (n = 502) | 1 (n = 306) | ≥2 (n = 115) |  |
| **Demographics** |  |  |  |  |  |  |  |
| Age, year | 73.0 ± 6.0 | 75.4 ± 6.4 | **< 0.001** | 72.6 ± 5.7 | 73.9 ± 6.2^*^ | 76.6 ± 6.8^*†^ | **< 0.001** |
| Female gender | 248 (35.5%) | 99 (44.0%) | **0.023** | 181 (36.1%) | 114 (37.3%) | 52 (45.2%) | 0.185 |
| Body mass index |  |  | 0.235 |  |  |  | **< 0.001** |
| <18.5 kg/m^2^ | 40 (5.7%) | 18 (8.0%) |  | 2 (0.4%) | 28 (9.2%)^*^ | 28 (24.3%)^*†^ |  |
| 18.5-23.9 kg/m^2^ | 365 (52.3%) | 102 (45.3%) |  | 269 (53.6%) | 143 (46.7%) | 55 (47.8%) |  |
| 24-27.9 kg/m^2^ | 234 (33.5%) | 81 (36.0%) |  | 181 (36.1%) | 107 (35.0%) | 27 (23.5%)^*^ |  |
| ≥28 kg/m^2^ | 59 (8.5%) | 24 (10.7%) |  | 50 (10.0%) | 28 (9.2%) | 5 (4.3%) |  |
| **General status** |  |  |  |  |  |  |  |
| ASA class |  |  | **< 0.001** |  |  |  | **< 0.001** |
| I | 7 (1.0%) | 0 (0.0%) |  | 6 (1.2%) | 1 (0.3%) | 0 (0.0%) |  |
| II | 477 (68.3%) | 60 (26.7%) |  | 366 (72.9%) | 134 (43.8%)^*^ | 37 (32.2%)^*^ |  |
| III | 208 (29.8%) | 153 (68.0%) |  | 126 (25.1%) | 161 (52.6%)^*^ | 74 (64.3%)^*^ |  |
| IV | 6 (0.9%) | 12 (5.3%) |  | 4 (0.8%) | 10 (3.3%)^*^ | 4 (3.5%) |  |
| Modified frailty index |  |  | --- |  |  |  | **< 0.001** |
| 0.00 | 220 (23.8%) | --- |  | 167 (33.3%) | 45 (14.7%)^*^ | 8 (7.0%)^*^ |  |
| 0.09 | 284 (30.8%) | --- |  | 197 (39.2%) | 74 (24.2%)^*^ | 13 (11.3%)^*†^ |  |
| 0.18 | 194 (21.0%) | --- |  | 138 (27.5%) | 41 (13.4%)^*^ | 15 (13.0%)^*^ |  |
| 0.27 | --- | 75 (11.3%) |  | 0 (0.0%) | 81 (26.5%)^*^ | 36 (31.3%)^*^ |  |
| 0.36 | --- | 41 (6.2%) |  | 0 (0.0%) | 48 (15.7%)^*^ | 27 (23.5%)^*^ |  |
| 0.45 | --- | 9 (1.4%) |  | 0 (0.0%) | 11 (3.6%)^*^ | 12 (10.4%)^*†^ |  |
| 0.55 | --- | 3 (0.5%) |  | 0 (0.0%) | 5 (1.6%)^*^ | 2 (1.7%)^*^ |  |
| 0.64 | --- | 1 (0.2%) |  | 0 (0.0%) | 1 (0.3%) | 2 (1.7%)^*^ |  |
| Malnutrition ^a^ | 153 (21.9%) | 59 (26.2%) | 0.182 | 0 (0.0%) | 126 (41.2%)^*^ | 86 (74.8%)^*†^ | **< 0.001** |
| **Comorbidities and history** ^b^ |  |  |  |  |  |  |  |
| Asthma | 15 (2.1%) | 4 (1.8%) | 0.943 | 14 (2.8%) | 1 (0.3%)^*^ | 4 (3.5%) | **0.030** |
| Obstructive sleep apnea ^c^ | 29 (4.2%) | 13 (5.8%) | 0.310 | 17 (3.4%) | 17 (5.6%) | 8 (7.0%) | 0.149 |
| Severe arrhythmia ^d^ | 45 (6.4%) | 32 (14.2%) | **< 0.001** | 29 (5.8%) | 39 (12.7%)^*^ | 9 (7.8%) | **0.002** |
| Other cardiac diseases ^e^ | 19 (2.7%) | 5 (2.2%) | 0.682 | 13 (2.6%) | 6 (2.0%) | 5 (4.3%) | 0.390 |
| Mental disorders ^f^ | 16 (2.3%) | 5 (2.2%) | 0.951 | 11 (2.2%) | 6 (2.0%) | 4 (3.5%) | 0.637 |
| Major neurodegenerative diseases ^g^ | 9 (1.3%) | 7 (3.1%) | 0.127 | 4 (0.8%) | 6 (2.0%) | 6 (5.2%)^*^ | **0.004** |
| Visual/hearing impairment | 21 (3.0%) | 10 (4.4%) | 0.299 | 16 (3.2%) | 8 (2.6%) | 7 (6.1%) | 0.201 |
| Chronic renal insufficiency ^h^ | 16 (2.3%) | 15 (6.7%) | **0.002** | 12 (2.4%) | 9 (2.9%) | 10 (8.7%)^*^ | **0.003** |
| Chronic hepatic dysfunction ^i^ | 36 (5.2%) | 13 (5.8%) | 0.718 | 21 (4.2%) | 14 (4.6%) | 14 (12.2%)^*^ | **0.002** |
| Hyper-/hypothyroidism | 13 (1.9%) | 6 (2.7%) | 0.639 | 8 (1.6%) | 9 (2.9%) | 2 (1.7%) | 0.411 |
| Chronic corticosteroid therapy ^j^ | 17 (2.4%) | 9 (4.0%) | 0.217 | 12 (2.4%) | 12 (3.9%) | 2 (1.7%) | 0.335 |
| Malignant tumor | 546 (78.2%) | 195 (86.7%) | **0.006** | 369 (73.5%) | 271 (88.6%)^*^ | 101 (87.8%)^*^ | **< 0.001** |
| Current smoker/quit ≤4 weeks ^k^ | 106 (15.2%) | 28 (12.4%) | 0.310 | 75 (14.9%) | 46 (15.0%) | 13 (11.3%) | 0.579 |
| Current alcoholism/quit ≤4 weeks ^l^ | 33 (4.7%) | 9 (4.0%) | 0.649 | 22 (4.4%) | 16 (5.2%)) | 4 (3.5%) | 0.719 |
| **Laboratory tests** |  |  |  |  |  |  |  |
| Hemoglobin <90 g/L | 51 (7.3%) | 31 (13.8%) | **0.003** | 0 (0.0%) | 27 (8.8%)^*^ | 55 (47.8%)^*†^ | **< 0.001** |
| Albumin ≤30 g/L | 32 (4.6%) | 12 (5.3%) | 0.647 | 0 (0.0%) | 7 (2.3%)^*^ | 37 (32.2%)^*†^ | **< 0.001** |
| Na^+^ <135.0 mmol/L | 80 (11.5%) | 36 (16.0%) | 0.074 | 49 (9.8%) | 34 (11.1%) | 33 (28.7%)^*^ | **< 0.001** |
| Ca^++^ <2.1 mmol/L | 17 (2.4%) | 15 (6.7%) | **0.003** | 12 (2.4%) | 14 (4.6%) | 6 (5.2%) | 0.141 |
| K^+^ <3.5 or >5.5 mmol/L | 82 (11.7%) | 17 (7.6%) | 0.077 | 59 (11.8%) | 30 (9.8%) | 10 (8.7%) | 0.517 |
| **Intraoperative factors** |  |  |  |  |  |  |  |
| Type of surgery |  |  | 0.871 |  |  |  | **< 0.001** |
| Simple general surgeries ^m^ | 63 (9.0%) | 17 (7.6%) |  | 61 (12.2%) | 17 (5.6%)^*^ | 2 (1.7%)^*^ |  |
| Gastric | 117 (16.8%) | 37 (16.4%) |  | 66 (13.1%) | 62 (20.3%)^*^ | 26 (22.6%)^*^ |  |
| Intestinal | 420 (60.2%) | 136 (60.4%) |  | 312 (62.2%) | 182 (59.5%) | 62 (53.9%) |  |
| Hepatopancreatobiliary | 98 (14.0%) | 35 (15.6%) |  | 63 (12.5%) | 45 (14.7%) | 25 (21.7%)^*^ |  |
| Surgery by Operative Stress Score ^n^ |  |  | 0.659 |  |  |  | **< 0.001** |
| Low stress | 46 (6.6%) | 10 (4.4%) |  | 45 (9.0%) | 10 (3.3%)^*^ | 1 (0.9%)^*^ |  |
| Moderate stress | 199 (28.5%) | 67 (29.8%) |  | 156 (31.1%) | 80 (26.1%) | 30 (26.1%) |  |
| High stress | 398 (57.0%) | 128 (56.9%) |  | 268 (53.4%) | 189 (61.8%) | 69 (60.0%) |  |
| Very high stress | 55 (7.9%) | 20 (8.9%) |  | 33 (6.6%) | 27 (8.8%) | 15 (13.0%) |  |
| Duration of surgery, hour | 3.2 (2.4, 4.3) | 3.0 (2.2, 4.1) | 0.286 | 3.1 (2.2, 4.1) | 3.2 (2.5, 4.3)^*^ | 3.4 (2.3, 4.6)^*^ | **0.004** |
| Type of anesthesia |  |  | 0.981 |  |  |  | 0.871 |
| General | 341 (48.9%) | 107 (47.6%) |  | 245 (48.8%) | 149 (48.7%) | 54 (47.0%) |  |
| Combined PNB-general | 329 (47.1%) | 109 (48.4%) |  | 14 (2.8%) | 12 (3.9%) | 6 (5.2%) |  |
| Combined epidural-general | 24 (3.4%) | 8 (3.6%) |  | 240 (47.8%) | 144 (47.1%) | 54 (47.0%) |  |
| Neuraxial | 4 (0.6%) | 1 (0.4%) |  | 3 (0.6%) | 1 (0.3%) | 1 (0.9%) |  |
| Seniority of anesthesiologists |  |  | 0.585 |  |  |  | 0.154 |
| <5 years | 197 (28.2%) | 62 (27.6%) |  | 143 (28.5%) | 93 (30.4%) | 23 (20.0%) |  |
| 5 to 10 years | 137 (19.6%) | 38 (16.9%) |  | 88 (17.5%) | 58 (19.0%) | 29 (25.2%) |  |
| >10 years | 364 (52.1%) | 125 (55.6%) |  | 271 (54.0%) | 155 (50.7%) | 63 (54.8%) |  |
| Blood transfusion | 52 (7.4%) | 12 (5.3%) | 0.277 | 23 (4.6%) | 25 (8.2%) | 16 (13.9%)^*^ | **0.001** |
| Estimated blood loss, ml | 100 (50, 200) | 100 (50, 200) | 0.594 | 60 (50, 200) | 100 (50, 200) | 100 (50, 200) ^*^ | **0.002** |

Data are n (%), mean ± SD, or median (interquartile range). *P* values in bold indicate <0.05. ^*^*P* <0.05/3 = 0.017 (Bonferroni-corrected *post hoc* multiple comparisons) when compared with the patients with a composite risk index of 0. ^†^*P* <0.017 when compared with the patients with a composite risk index of 1.

*ASA* American Society of Anesthesiologists, *PNB* peripheral nerve block

^a^ Defined by any of the following: (1) a [body mass index](https://www.ncbi.nlm.nih.gov/books/n/nicecg32/glossary/def-item/glossary.gl1-d20/) of less than 18.5 kg/m^2^; (2) unintentional weight loss of greater than 10% within the last 3–6 months; or (3) a body mass index of less than 20 kg/m^2^ and unintentional weight loss of greater than 5% within the last 3–6 months [33].

^b^ Data on 11 items of the modified frailty index are presented in Supplementary Table 1.

^c^ Diagnosed by previous polysomnography, or history inquiry and physical examination, and/or STOP-Bang/Berlin questionnaire.

^d^ Include atrial fibrillation, frequent (>6 beats/min) or multifocal ventricular premature beat, paroxysmal supraventricular tachycardia, second/third-degree atrioventricular block, and sick sinus syndrome.

^e^ Include congenital heart disease, cardiomyopathy, and valvular heart disease.

^f^ Include diagnosed depression, anxiety, schizophrenia, phobia, and hallucination.

^g^ Include Alzheimer's disease, Parkinson's disease, and dementia.

^h^ Refers to estimated glomerular filtration rate <45 ml/min/1.73 m² or on dialysis [37]. The CKD-EPI equation was adopted to calculate the estimated glomerular filtration rate [38].

^i^ Defined as Child-Pugh class B and C.

^j^ With a duration of >1 month.

^k^ Smoking refers to daily smoking of cigarettes up to half a pack for at least two years.

^l^ Alcoholism refers to ethanol consumption ≥40 g/d for men and ≥20 g/d for women, lasting for more than 5 years. Ethanol (g) = alcohol consumption (ml) × ethanol content (%) × 0.8 [32].

^m^ Refers to low-risk and 23-hour-stay operations including hernia repair, laparoscopic cholecystectomy, appendectomy, and hepatic cyst fenestration.

^n^ Stratified into five categories of physiologic stress, i.e., very low stress, low stress, moderate stress, high stress, and very high stress [34]. Also see Supplementary Table 2.
