# Supplementary material for: The composite risk index based on frailty predicts postoperative complications in older patients recovering from elective digestive tract surgery: a retrospective cohort study
Source: BMC Anesthesiol. 2022 Jan 3;22:7. doi: 10.1186/s12871-021-01549-6 (PMC8722296; doi:10.1186/s12871-021-01549-6)
Supplement: Supplementary file 6 — Additional file 6: Supplementary Table 6 Factors in association with postoperative complications (univariate analyses). [file 12871_2021_1549_MOESM6_ESM.docx]

**Supplementary Table 6** Factors in association with postoperative complications (univariate analyses)

| Variable | Number | Odds ratio (95% CI) | *P* value |
| --- | --- | --- | --- |
| **Demographics** |  |  |  |
| Age, year | 923 | 1.020 (0.997-1.044) | **0.092** |
| Female gender | 347 | 1.183 (0.881-1.589) | 0.263 |
| Body mass index |  |  |  |
| <18.5 kg/m^2^ | 58 | Reference |  |
| 18.5-23.9 kg/m^2^ | 467 | 0.456 (0.261-0.799) | **0.006** |
| 24-27.9 kg/m^2^ | 315 | 0.512 (0.288-0.909) | **0.022** |
| ≥28 kg/m^2^ | 83 | 0.537 (0.266-1.085) | **0.083** |
| **General status** |  |  |  |
| American Society of Anesthesiologists class |  |  |  |
| I/II | 544 | Reference |  |
| III | 361 | 2.412 (1.789-3.253) | **<0.001** |
| IV | 18 | 7.982 (2.930-21.75) | **<0.001** |
| Modified frailty index ^a^ |  |  |  |
| 0.00 | 220 | Reference |  |
| 0.09 | 284 | 1.173 (0.759-1.813) | 0.471 |
| 0.18 | 194 | 1.629 (1.032-2.571) | **0.036** |
| 0.27 | 117 | 2.305 (1.393-3.815) | **0.001** |
| 0.36 | 75 | 3.413 (1.943-5.998) | **<0.001** |
| ≥0.45 | 33 | 6.333 (2.921-13.73) | **<0.001** |
| Malnutrition ^b^ | 212 | 2.041 (1.476-2.822) | **<0.001** |
| **Comorbidities and history** ^c^ |  |  |  |
| Asthma | 19 | 1.201 (0.451-3.194) | 0.714 |
| Obstructive sleep apnea ^d^ | 42 | 3.347 (1.790-6.258) | **<0.001** |
| Severe arrhythmia ^e^ | 77 | 1.638 (1.008-2.661) | **0.046** |
| Other cardiac diseases ^f^ | 24 | 1.575 (0.680-3.645) | 0.289 |
| Mental disorders ^g^ | 21 | 1.304 (0.520-3.269) | 0.571 |
| Major neurodegenerative diseases ^h^ | 16 | 3.416 (1.259-9.273) | **0.016** |
| Visual/hearing impairment ^i^ | 31 | 1.918 (0.926-3.974) | **0.080** |
| Chronic renal insufficiency ^j^ | 31 | 2.199 (1.068-4.530) | **0.033** |
| Chronic hepatic dysfunction ^k^ | 49 | 2.216 (1.237-3.967) | **0.007** |
| Hyper-/hypothyroidism | 19 | 0.924 (0.329-2.592) | 0.881 |
| Chronic corticosteroid therapy ^l^ | 26 | 1.645 (0.736-3.673) | 0.225 |
| Malignant tumor ^m^ | 741 | 2.238 (1.468-3.411) | **<0.001** |
| Current smoker/quit ≤4 weeks ^n^ | 134 | 1.170 (0.784-1.744) | 0.442 |
| Current alcoholism/cessation ≤4 weeks ^o^ | 42 | 1.313 (0.680-2.536) | 0.418 |
| **Laboratory tests** |  |  |  |
| Hemoglobin <90 g/L | 82 | 2.894 (1.827-4.582) | **<0.001** |
| Albumin ≤30 g/L | 44 | 3.327 (1.804-6.136) | **<0.001** |
| Na^+^ <135.0 mmol/L | 116 | 1.139 (0.744-1.743) | 0.550 |
| Ca^++^ <2.1 mmol/L | 32 | 1.373 (0.653-2.890) | 0.403 |
| K^+^ <3.5 or >5.5 mmol/L | 99 | 1.083 (0.684-1.714) | 0.734 |
| **Composite risk index** ^p^ |  |  |  |
| 0 | 502 | Reference |  |
| 1 | 306 | 2.604 (1.878-3.612) | **<0.001** |
| 2 | 92 | 3.898 (2.437-6.236) | **<0.001** |
| ≥3 | 23 | 10.61 (4.239-26.54) | **<0.001** |
| **Intraoperative factors** |  |  |  |
| Type of surgery |  |  |  |
| Simple general surgeries ^q^ | 80 | Reference |  |
| Gastric | 154 | 13.860 (4.175-46.015) | **<0.001** |
| Intestinal | 556 | 8.804 (2.735-28.338) | **<0.001** |
| Hepatopancreatobiliary | 133 | 19.849 (5.959-66.116) | **<0.001** |
| Surgery type by Operative Stress Score ^r^ |  |  |  |
| Low stress | 56 | Reference |  |
| Moderate stress | 266 | 13.05 (1.764-96.51) | **0.012** |
| High stress | 526 | 26.04 (3.573-189.7) | **0.001** |
| Very high stress | 75 | 50.77 (6.675-386.1) | **<0.001** |
| Duration of surgery, hour | 923 | 1.312 (1.200-1.434) | **<0.001** |
| Type of anesthesia |  |  |  |
| General | 448 | Reference |  |
| Regional/combined regional-general ^s^ | 475 | 0.952 (0.714-1.270) | 0.740 |
| Seniority of anesthesiologists |  |  |  |
| <5 years | 259 | Reference |  |
| 5-10 years | 175 | 1.262 (0.822-1.938) | 0.288 |
| >10 years | 489 | 1.197 (0.850-1.686) | 0.303 |
| Estimated blood loss, 100 ml | 923 | 1.094 (1.045-1.146) | **<0.001** |
| Intraoperative blood transfusion | 64 | 2.631 (1.575-4.396) | **<0.001** |

*P* values in bold indicate <0.20.

^a^ Each item of 11 frailty components was assigned the same weight of 1 point. The modified frailty index score was calculated by confirming the total points of each patient and then dividing the total points by 11 [7].

^b^ Defined by any of the following: (1) a [body mass index](https://www.ncbi.nlm.nih.gov/books/n/nicecg32/glossary/def-item/glossary.gl1-d20/) of less than 18.5 kg/m^2^; (2) unintentional weight loss of greater than 10% within the last 3–6 months; (3) a body mass index of less than 20 kg/m^2^ and unintentional weight loss of greater than 5% within the last 3–6 months [33].

^c^ The 11 components of modified frailty index were excluded.

^d^ Diagnosed by previous polysomnography, or by consulting anesthesiologists or otolaryngologists according to history inquiry, physical examination, and/or STOP-Bang/Berlin questionnaire.

^e^ Include atrial fibrillation, frequent (>6 beats/min) or multifocal ventricular premature beat, paroxysmal supraventricular tachycardia, second/third degree atrioventricular block, and sick sinus syndrome.

^f^ Include congenital heart disease, cardiomyopathy, and valvular heart disease.

^g^ Include diagnosed depression, anxiety, schizophrenia, phobia, and hallucination.

^h^ Include Alzheimer disease, Parkinson's disease, and dementia.

^i^ Visual impairment includes blindness, poor vision, or visual field damage caused by various diseases such as cataract, glaucoma, trauma, which affect daily living. Hearing impairment includes permanent hearing defect induced by any cause, with an inability to hear or muffled hearing of ambient or speech sounds.

^j^ Refers to estimated glomerular filtration rate (eGFR) <45 ml/min/1.73 m² or on dialysis [37]. The CKD-EPI equation was adopted to calculate the eGFR. Female: eGFR=144×(Scr/0.7)-0.329×(0.993) ×Age when Scr ≤62 μmol/L, eGFR=144×(Scr/0.7)-1.209×(0.993) × Age when Scr >62 μmol/L; male: eGFR=144×(Scr/0.9)-0.411×(0.993) × Age when Scr ≤ 80 μmol/L, eGFR=144×(Scr/0.9)-1.209×(0.993) ×Age when Scr >80 μmol/L [38].

^k^ Defined as Child-Pugh class B and C.

^l^ With a duration of >1 month.

^m^ Confirmed by postoperative pathological examination.

^n^ Smoking refers to daily smoking of cigarettes up to half a pack for at least two years.

^o^ Alcoholism refers to ethanol consumption ≥ 40 g/d for men and ≥ 20 g/d for women, lasting for more than 5 years. Ethanol (g) = alcohol consumption (ml) × ethanol content (%) × 0.8 [32].

^p^ A composite of four items, i.e., modified frailty index ≥0.27, malnutrition [33], moderate or severe anemia (hemoglobin <90 g/L), and severe hypoalbuminemia (albumin ≤30 g/L). Each item was assigned the same weight of 1 point.

^q^ Refers to low-risk and 23-hour-stay operations including hernia repair, laparoscopic cholecystectomy, appendectomy, and hepatic cyst fenestration.

^r^ Stratified into five categories of physiologic stress, i.e., very low stress, low stress, moderate stress, high stress, and very high stress [34]. Also see Supplementary Table 2.

^s^ Includes combined peripheral nerve block-general anesthesia, combined epidural-general anesthesia, and neuraxial anesthesia.
