# Supplementary figures and images for: The composite risk index based on frailty predicts postoperative complications in older patients recovering from elective digestive tract surgery: a retrospective cohort study
Source: BMC Anesthesiol. 2022 Jan 3;22:7. doi: 10.1186/s12871-021-01549-6 (PMC8722296; doi:10.1186/s12871-021-01549-6)

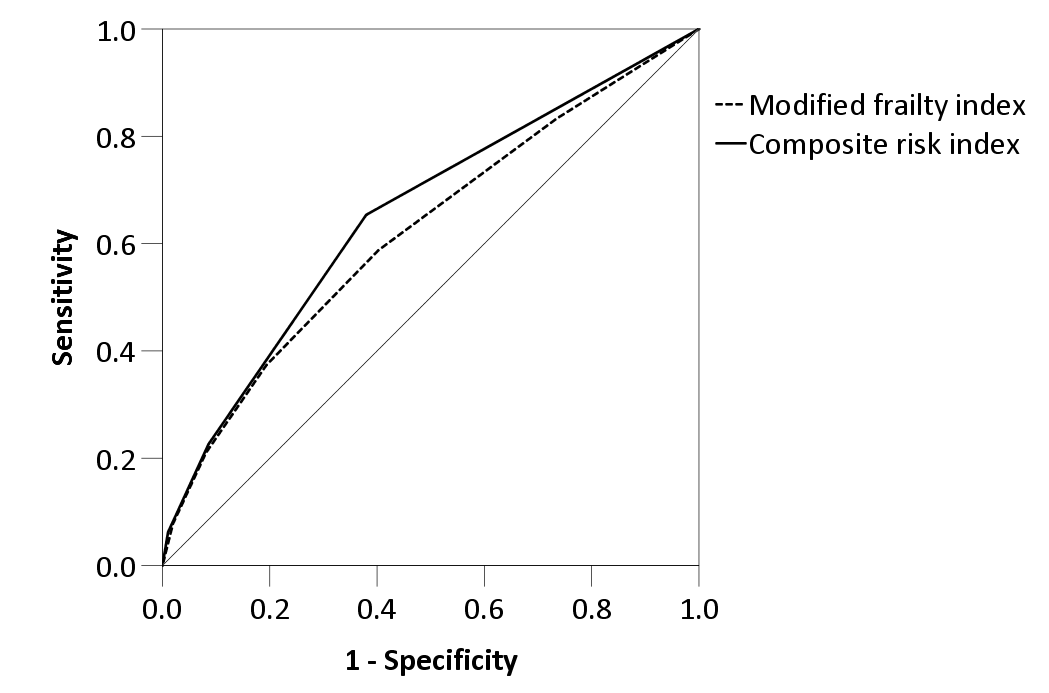

Supplement: Supplementary file 7 — Additional file 7: Supplementary Figure 1 The area under receiver-operator characteristic curves of modified frailty index and composite risk index in predicting postoperative complications. [file 12871_2021_1549_MOESM7_ESM.docx]
